# Caveolin 3, Flotillin 1 and Influenza Virus Hemagglutinin Reside in Distinct Domains on the Sarcolemma of Skeletal Myofibers

**DOI:** 10.1155/2012/497572

**Published:** 2012-03-05

**Authors:** Mika Kaakinen, Tuula Kaisto, Paavo Rahkila, Kalervo Metsikkö

**Affiliations:** ^1^Department of Anatomy and Cell Biology, Institute of Biomedicine, University of Oulu, P.O. Box 5000, Aapistie 7, 90014 Oulu, Finland; ^2^Department of Health Sciences, University of Jyväskylä, P.O. Box 35, 40014 Jyväskylä, Finland

## Abstract

We examined the distribution of selected raft proteins on the sarcolemma of skeletal myofibers and the role of cholesterol environment in the distribution. Immunofluorescence staining showed that flotillin-1 and influenza hemagglutinin exhibited rafts that located in the domains deficient of the dystrophin glycoprotein complex, but the distribution patterns of the two proteins were different. Cholesterol depletion from the sarcolemma by means of methyl-**β**-cyclodextrin resulted in distorted caveolar morphology and redistribution of the caveolin 3 protein. Concomitantly, the water permeability of the sarcolemma increased significantly. However, cholesterol depletion did not reshuffle flotillin 1 or hemagglutinin. Furthermore, a hemagglutinin variant that lacked a raft-targeting signals exhibited a similar distribution pattern as the native raft protein. These findings indicate that each raft protein exhibits a strictly defined distribution in the sarcolemma. Only the distribution of caveolin 3 that binds cholesterol was exclusively dependent on cholesterol environment.

## 1. Introduction

Skeletal myofibers are unique cells that have a large plasma membrane separated into sarcolemma and transverse tubules. The dystrophin glycoprotein complex (DGC) distributes at the sarcolemma in an organized fashion forming a cross-striated pattern over the I-bands and M-lines of the underlying myofibrils and longitudinal stripes over the I-A junctional areas [1, 2]. DGC is thought to stabilize the sarcolemmal membrane that is subject to mechanical stress during muscle contractions. Accordingly, inherited defects in the components of the DGC and especially in dystrophin are manifested in various types of dystrophia diseases [3]. Interestingly, many endogenous sarcolemmal proteins such as Na,K-ATPase [2], transferrin receptor [4], caveolin 3 (cav 3) [4], chloride channel ClC-1 [5], and aquaporin 4 [6] occupy the areas covered by the DGC. Caveolae pit structures that contain cav 3 protein also locate to the DGC regions. Several viral model glycoproteins also show a specific localization pattern in relation to the DGC mosaic [4].

Studies with mononucleated cells and giant plasma membrane vesicles have revealed that caveolins and several lipid-anchored proteins show a strong preference to cholesterol and sphingolipid-enriched lipid islets called rafts [7, 8]. These microdomains have been reported to exist as dispersed units of nanometer scale, but they may also form large clusters of several micrometers in size that lack nonraft proteins [9–12]. The integrity of rafts is crucially dependent on cholesterol. Accordingly, cholesterol depletion by a cholesterol-sequestering drug, methyl-*β*-cyclodextrin (CDX), inhibits patching of antibody cross-linked raft proteins and increases the diffusion rates of certain raft-associated proteins [10, 13, 14].

Since the sarcolemma of myofibers comprises a regularly repeated two-domain mosaic and sarcolemmal nonraft proteins have been shown to comply with this pattern, we examined here whether the distribution patterns of raft proteins were domain specific. We found that contrasting the behavior of cav 3, flotillin 1, and the classical raft marker influenza virus hemagglutinin (HA) localized into the domains deficient of DGC. We next investigated whether the cholesterol environment affected the distribution of raft proteins between the sarcolemmal domains. Interestingly, the localization pattern of the caveolar protein cav 3 was altered upon cholesterol depletion, and the caveolar pits were deformed or destroyed. However, depletion of cholesterol did not reshuffle flotillin 1 or HA. Furthermore, removal of the raft-targeting signals from HA indicated that the domain-specific localization was not dependent on the raft-targeting signals. While cholesterol was important to locate cav 3 into caveolae, it did not affect the distribution of flotillin 1 or HA on the sarcolemma.

## 2. Methods

### 2.1. Isolation and Cultivation of Myofibers

Myofibers were isolated from the flexor digitorum brevis (FDB) muscle of three-month-old female rats by using collagenase digestion as described [15]. The isolated myofibers were mounted on cell culture dishes coated with Matrigel (Becton-Dickinson Biosciences, Franklin Lake, NJ, USA) and cultivated in minimal essential medium (MEM) supplemented with 5% horse serum, L-glutamine and penicillin-streptomycin. For measurements of live cells, the isolated myofibers were mounted on Matrigel-coated glass-bottom dishes (WillcoWells, Amsterdam, Netherlands).

### 2.2. CDX Treatments

Treatments with CDX (Sigma-Aldrich, St. Louis, Mo. USA) were performed in MEM containing L-glutamine, penicillin-streptomycin, and 0.05% (v/v) heat-inactivated and lipoprotein-depleted horse serum. The serum was delipidified by sequential ultracentrifugation prior to use to remove lipoprotein components, as described by Goldstein et al. [16]. Prior to the CDX treatments, the myofiber cultures were washed two times with MEM. The fibers were incubated in the presence of appropriate CDX concentrations in a volume of 0.5 mL for 1 h at 37°C.

### 2.3. Detergent Extractions

Isolated myofibers were scraped from the culture dishes and pelleted by centrifugation at 200 g for 2 min in a table top centrifuge. After that, the myofiber pellets were suspended in cold phosphate buffered saline (PBS) containing 1% Triton X-100 and complete protease inhibitor cocktail (Roche, Basel, Switzerland) and incubated for 10 min in an ice bath. The detergent-insoluble proteins were pelleted by centrifugation at 75,000 g for 2.5 h at 4°C. Proteins in the supernatants were precipitated with 10% TCA. Proteins in the pellets and the supernatants were separated with SDS/PAGE followed by western blotting using rabbit antiflotillin 1 (Sigma-Aldrich) as primary antibody and peroxidase-conjugated anti-rabbit IgG (Bio-Rad Laboratories) as secondary antibodies. Detection was with chemiluminescence detection reagent (GE Healthcare) using Hyperfilm (GE Healthcare).

### 2.4. Hypotonic Swelling Experiments

Freshly isolated myofibers on glass-bottom dishes were either treated or not treated with CDX and then incubated in the presence of 10 mM fluorophore Calcein-AM (Invitrogen, Eugene, OR, USA) for 5 min at 37°C. Thereafter, the culture was washed two times with MEM and covered with an additional layer of Matrigel to prevent detachment of myofibers during subsequent treatments as described in [6]. The myofibers were examined with Zeiss LSM510 confocal microscope (Carl Zeiss, Göttingen, Germany), and those showing a homogenous fluorescence were chosen for measurements. The region of interest was the entire myofiber, and the focal plane was set at the core region. A 60 s recording was performed before the hypotonic shock that was induced by changing isotonic PBS (300 mosM) to hypotonic (150 mosM). Changing the medium resulted in a variable amount of movement of the myofiber being measured. Therefore, the region of interest was manually repositioned to correspond to the new location of the myofiber. All the recordings were performed at 23 ± 1°C by measuring the mean fluorescence intensity at 2 s intervals. The fluorescence intensity changes were analyzed by using Zeiss LSM510 Pascal software, and the measurement covered the entire fiber. Since a small fraction of the FDB myofibers contains aquaporin 4 [6], the presence of aquaporin 4 was determined by immunofluorescence staining after the recordings. Measurements from myofibers containing aquaporin 4 were rejected since aquaporin 4 is a water channel.

### 2.5. Electron Microscopy

For immunolocalization studies, extensor digitorum longus (EDL) muscle was dissected and fixed with 4% paraformaldehyde in 0.1 M phosphate buffer for 1 h, sliced in small pieces and placed in 2.3 M sucrose overnight. Alternatively, isolated FDB myofibers were fixed with 4% paraformaldehyde for 1 h in 0.1 M phosphate buffer containing 73 mM sucrose, and then detached from the culture dishes and centrifuged at 2000 g for 1 min. After washings with PBS, the pellets were suspended in 12% gelatin at 37°C, centrifuged at 3000 g for 5 min, and chilled in an ice bath for 30 min. The congealed gelatin was sliced in small pieces and placed in 2.3 M sucrose overnight. The pieces of EDL or the gelatin-embedded FDB myofibers were frozen with liquid nitrogen, and thin sections (200 nm) were cut. The sections were first incubated in 50 mM glycine in PBS and then in 5% bovine serum albumin (BSA) supplemented with 0.1% cold water fish skin gelatin (Aurion, Wageningen, Netherlands) in PBS to block nonspecific binding. Antibodies were diluted with 0.1% BSA-C (Aurion) in PBS, and the incubations were for 1 h at 37°C. Rabbit antimouse secondary antibodies (Zymed Laboratories) and protein A-gold complex [17] were used for detection. Sections were examined with Philips CM100 transmission electron microscope.

For conventional transmission electron microscopy, the isolated myofibers were fixed with 1.5% glutaraldehyde in PBS. The fixed fibers were scraped and pelleted by centrifugation and immersed in 12% agarose. Osmium tetroxide (1%) was used for postfixation, and the myofibers were embedded in Epon LX 112 (Ladd Research Industries, Burlington, Vt. USA). Thin sections (150 nm) were examined with the Philips CM100 electron microscope. The number of caveolae/*μ*m of the sarcolemma was calculated using 4–7 photographed fields/fiber. Image analysis was performed with UTHSCSA Image Tool for Windows version 3.

### 2.6. Recombinant Viruses and *In Vitro* Mutagenesis

Preparation of recombinant Semliki Forest Virus (SFV) particles encoding HA Japan/A/305/57 [Genbank: DQ508841.1] has been described previously [18]. To generate SFV particles encoding a mutant HA lacking all known raft-targeting signals, we used the mutant 2A511 HA described by Scheiffele et al. [19]. The cDNA of the mutant 2A511 HA in pSFV vector was subjected to *in vitro* mutagenesis to change the triplets encoding cysteine 536 at the C-terminal end of the transmembrane domain and cysteines 543 and 546 in the C-terminal tail into triplets encoding serines, to prevent palmitoylation of the protein [7]. The *in vitro* mutagenesis was performed by using the QuickChange site directed *in vitro* mutagenesis kit (Stratagene, La Jolla, CA, USA). That the mutated product had the desired sequence was verified with ABI PRISM 3130XL sequencer and BigDye Terminator v1.1 Cycle Sequencing Kit (Applied Biosystems Inc., Foster City, CA, USA).

The isolated myofibers were infected with the recSFVs by applying viral stock medium into the culture medium at 1 : 3 dilution. The infection was allowed to proceed for 16–24 h at 37°C.

### 2.7. Immunohistochemistry

Isolated myofibers were fixed with 3% paraformaldehyde in PBS for 10 min. After permeabilization with 1% Triton X-100, the nonspecific binding was blocked with 1% BSA for 10 min. Primary antibodies were applied for 30 min at 37°C or 2 h at room temperature. The primary antibodies used were rabbit antiflotillin 1 (Sigma-Aldrich), mouse anti-*β*-dystroglycan (Novocastra Laboratories Ltd, Benton Lane, UK), mouse anti-cav 3 (Becton Dickinson, Franklin Lakes, NJ, USA), and mouse antidihydropyridine receptor (DHPR) (Affinity Bioreagents Inc., Golden, CO, USA). Secondary antibodies were Alexa 488-conjugated anti-rabbit IgG (Invitrogen) or Alexa 568-conjugated anti-mouse IgG (Invitrogen), and incubations lasted for 30 min at 37°C. HA on the sarcolemma was detected by adding rabbit anti-HA antiserum [19] into the culture medium at 1 : 100 dilution. Incubation was for 1.5 h at 10–12°C followed by two washes with PBS and fixation with paraformaldehyde. Then the Alexa 488-conjugated anti-rabbit IgG was applied and incubated for 30 min at 37°C. The myofibers were next permeabilized by a 5-minute treatment with 1% Triton X-100 in PBS and processed for double immunofluorescence staining for *β*-dystroglycan. Negative controls for all antibodies were performed by omitting the primary antibody, and these were blank. Samples were examined with Zeiss LSM510 confocal microscope.

### 2.8. Statistics

All the data are expressed as mean ± SD, and *n* indicates the number of determinations. Two-sample *t*-test was used to compare two groups, and one-tailed hypothesis testing was used to determine *P* values. *P* < 0.05 was considered statistically significant.

## 3. Results

### 3.1. Flotillin 1 and Cav 3 Reside in Separate Membrane Microdomains

**Figure 1:**
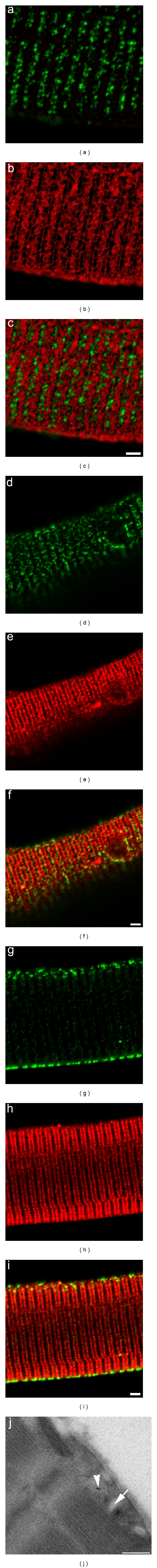
Flotillin 1 resides in the DGC-deficient regions in structures near transverse tubule openings. A confocal section at the sarcolemma level indicates that flotillin 1 (a) appears as spots of irregular shape. Double staining for *β*-dystroglycan (b) and the merged image (c) show that dystroglycan and flotillin 1 do not colocalize. A confocal section at the sarcolemma level indicated that flotillin 1 (d) and DHPR (e) that mark transverse tubules are flanking each other, demonstrated in the merged image (f). A longitudinal confocal section in the middle of a myofiber indicates that flotillin 1 (g) exhibits intense staining that seems to be an extension of the DHPR staining (h) as demonstrated in the merged image (i). Immunoelectron microscopy for flotillin 1 in EDL section shows gold particles (arrowhead) in a structure beneath the sarcolemma near the transverse tubule neck (arrow) (j). Scale bars, 2 *μ*m (a–i); 250 nm (j).

The flotillin rafts are distinct from caveolae in mononucleated cells [20] in which the flotillin microdomains can exist in either flat or invaginated state [21]. Here, we examined whether flotillin 1 microdomains in skeletal muscle cells were distinct from the caveolae that contain cav 3. For this purpose, we performed double immunofluorescence staining for the two proteins in isolated myofibers that provide a view over the muscle cell surface. Figures 1(a)–1(c) show that flotillin 1 appeared as clusters at the A-band regions in the domains deficient of DGC. These domains are lacking cav 3 [4]. 

Since we found flotillin 1 in the regions deficient of DGC that are known to harbor transverse tubule openings [4], we next performed double staining for flotillin 1 and the transverse tubule marker DHPR. Confocal sectioning indicated that flotillin 1 staining flanked that of the DHPR staining (Figures 1(d)–1(f)), suggesting that the two proteins were close to each other but did not overlap. A longitudinal confocal section through a myofiber (Figures 1(g)–1(i)) suggests that flotillin 1 marked structures in the transverse tubule neck portions. Supporting this, immunoelectron microscopy studies consistently indicated that flotillin 1 located in structures 50–100 nm beneath the sarcolemmal membrane.  Figure 1(j) shows an example.

### 3.2. Cholesterol Depletion Partially Reshuffles Cav 3 and Deforms Caveolae but Does Not Affect Flotillin 1 Distribution

**Figure 2:**
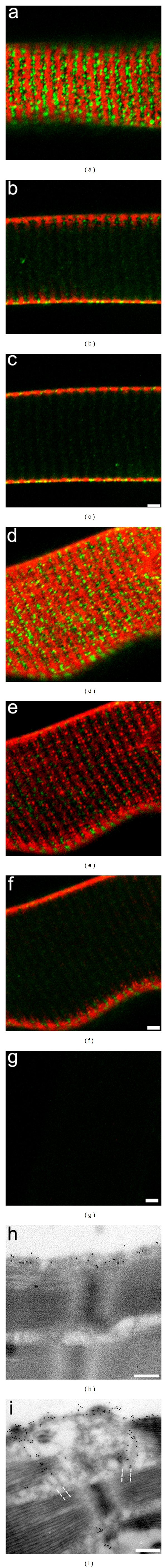
Cav 3 and flotillin 1 reside in different types of rafts. Cav 3 (red) normally exhibits a cross-striated distribution pattern over the I bands, while flotillin 1 (green) locates to the A-band regions lacking cav 3 (a). A confocal **s**ection 5 *μ*m beneath the sarcolemma shows neither flotillin 1 nor cav 3 (b). A confocal section at the middle of the myofiber shows flotillin and cav 3 on the sarcolemma exclusively (c). After treatment with 5 mM CDX, the sarcolemmal staining patterns retain (d), however, a confocal section 5 *μ*m beneath the sarcolemma of the CDX-treated myofiber reveals double rows of dots of cav 3 (e). Some subsarcolemmal dots are also visible in the middle section (f). Scale bars, 2 *μ*m. The flotillin and cav 3 staining patterns were abolished upon omitting the primary antibodies (g). EM immunogold labeling of FDB cryosections shows particles indicating the presence of cav 3 predominantly in the sarcolemma in myofibers not treated with CDX (h). In FDB myofiber treated with 5 mM CDX, the immunogold particles are associated with the transverse tubules, marked by dotted lines (i). Scale bar in (h, i) 250 nm.

We have previously reported that cav 3 disappears from the sarcolemma upon cholesterol depletion [6]. The finding that flotillin and cav 3 seem to reside in discrete structures prompted us to investigate whether depletion of cholesterol affected the distribution pattern of flotillin 1, too. We found that the immunofluorescence staining pattern of flotillin 1 remained unchanged after CDX treatment of the isolated myofibers. The intensity of flotillin immunostaining varied considerably from fiber to fiber; however, a systematic intensity change could not be observed upon CDX treatment. The intensity of the cav 3 staining on the sarcolemma was reduced in variable extent. A salient feature after the CDX treatment was that rows of spots of cav 3 staining appeared beneath the sarcolemma (Figure 2(e)). The staining pattern mimicked that of DHPR suggesting localization of cav 3 in transverse tubules. Immunogold labeling verified that after cholesterol depletion cav 3 was abundant especially at the neck portions of the transverse tubules. Figures 2(h)–2(i) summarize these results.

**Figure 3:**
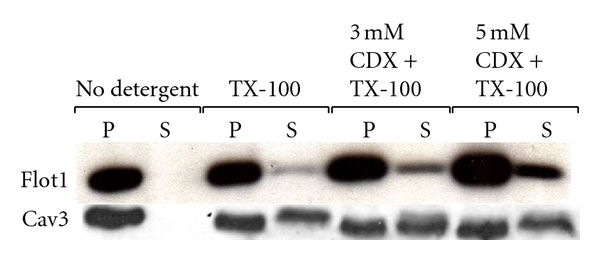
Flotillin 1 is more sparingly soluble in cold Triton X-100 than cav 3. Isolated myofibers were treated with 0, 3, and 5 mM CDX and then extracted with 1% Triton X-100. Soluble material (S) and pellets (Ps) were subjected to SDS/PAGE and western blotting using specific antibodies. Treatment of the myofibers with CDX only slightly increased the solubility of flotillin 1 to the detergent, whereas the solubility of cav 3 remained unaffected.

We next subjected cultured myofibers to various concentrations of CDX followed by extraction with cold Triton X-100. We found that flotillin 1 was sparingly soluble in Triton X-100 (soluble fraction was 21.5 ± 3.7%, *n* = 2), and surprisingly, CDX treatment only slightly increased its detergent solubility (3 mM CDX: 30.4 ± 5.6%, *n* = 3; 5 mM CDX: 31 ± 6.4%, *n* = 3). Similar analysis was also performed for cav 3, indicating that CDX treatment did not increase the solubility of the protein in Triton X-100.  Figure 3 shows an example of the results. Both flotillin 1 as well as cav 3 floated in sucrose gradients, indicating that the insolubility was due to association with rafts. These findings suggest that flotillin 1, like cav 3, resides in a very compactly packed lipid environment.

**Figure 4:**
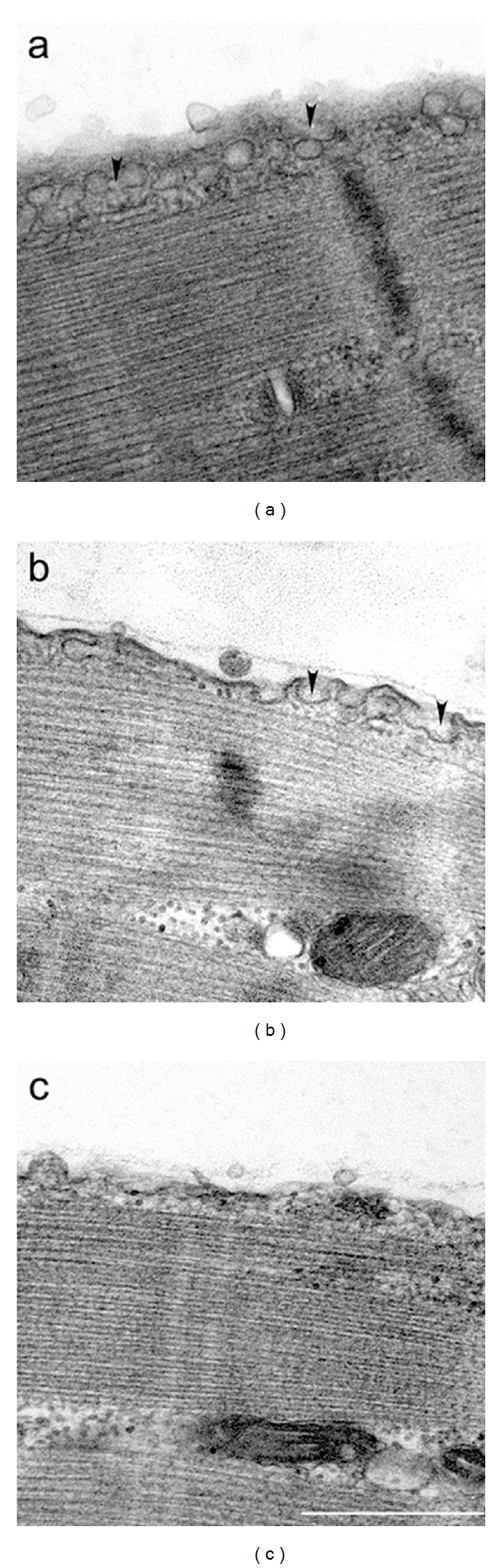
Cholesterol depletion destroys the morphology of caveolae. (a) In an intact FDB myofiber, there are abundantly flask-shaped caveolae that form rosettes (marked by arrowheads) beneath the sarcolemma. (b) Treatment with 1 mM CDX reduces the number of caveolae, and the morphology of individual caveolae (arrowheads) is distorted. (c) Treatment with 5 mM CDX results in flattened caveolae without any distinguishable morphology. Scale bar, 500 nm.

Since cav 3 disappears from the sarcolemma upon CDX treatment, we next examined whether caveolae pits disappeared. Transmission electron microscopy studies of myofibers after CDX treatment indicated that, in comparison to the normal morphology of caveolae (Figure 4(a)), deformation occurred at 1 mM concentration of the drug (Figure 4(b)). Furthermore, the number of caveolae was reduced by about 50% in CDX-treated myofibers (2.9 ± 0.34 caveola/*μ*m, *n* = 5 photographs) as compared to the controls without any drug treatment (5.9 ± 0.01 caveola/*μ*m, *n* = 2). Increasing the CDX concentration to 5 mM resulted in destruction of the caveolar morphology (Figure 4(c)). These findings are compatible with those obtained with nonmuscle cells [22].

**Figure 5:**
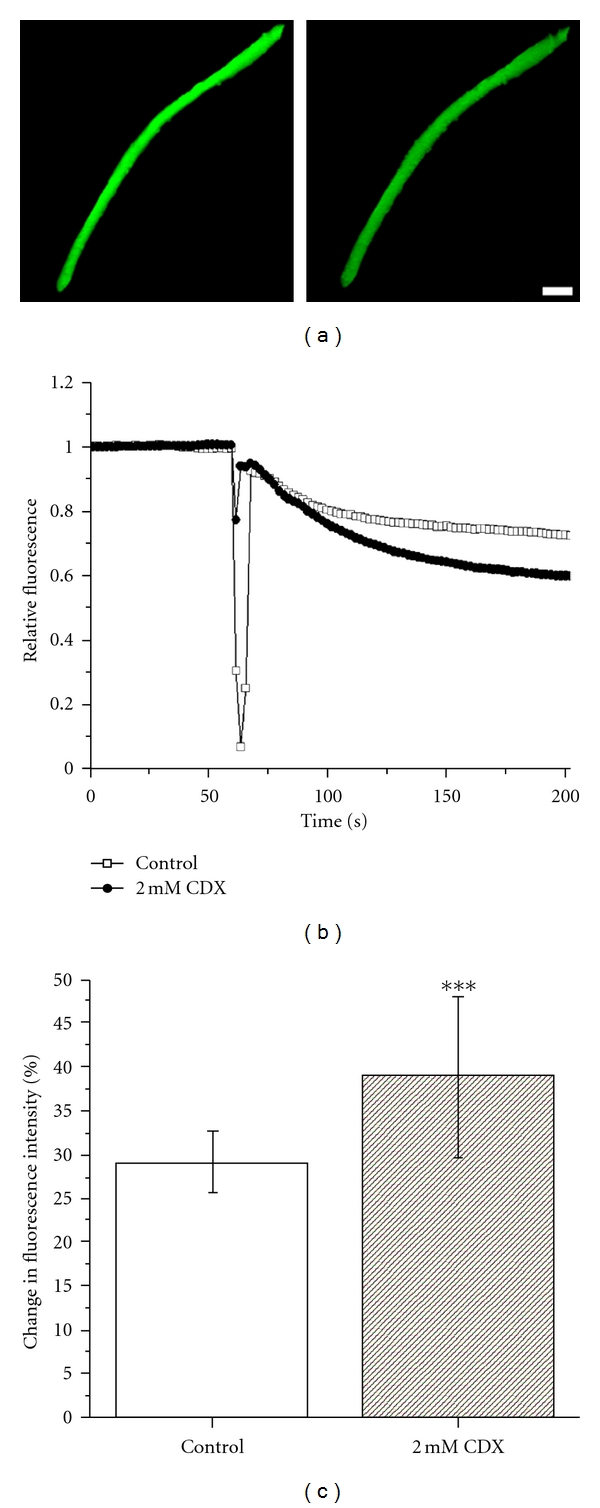
Cholesterol depletion increases the water permeability of isolated myofibers. (a) Fluorescence micrographs of a calcein-loaded myofiber before (left) and 140 s after switching to the hypotonic buffer (right) that indicates decreased fluorescence. The myofiber was not treated with CDX. Scale bar, 50 *μ*m. (b) Examples of the relative fluorescence (fluorescence at certain time point divided by the fluorescence at the beginning of the experiment) recordings for myofibers treated or not treated with CDX are shown. A sharp decline of the fluorescence intensity was often seen upon switching to the hypotonic buffer, owing to sample movement. (c) The change of fluorescence intensity is presented as a percentage between the value of fluorescence intensity in isotonic buffer just before and 140 s after switching from isotonic to hypotonic condition. *N* = 14 (control) and 16 (CDX-treated) myofibers from five different isolations. ***P < 0.001.

In addition to the morphology of caveolae, cholesterol has an impact on the water permeability of membranes [23]. This question is especially important with regard to the sarcolemma, owing to the fact that cholesterol-lowering medication has adverse effects on skeletal muscle. We therefore investigated whether the cholesterol depletion altered the swelling response of myofibers under hypotonic conditions. Interestingly, we found that after switching from isotonic to hypotonic osmolarity the intensity of calcein-AM reduced significantly more in the cholesterol-depleted myofibers as compared to control myofibers (Figure 5). This suggests that the sarcolemma became more permeable to water. The effect was repeatable with 2 mM CDX, but at higher drug concentrations, a major fraction of the myofibers became leaky for the fluorophore as indicated by a slight decrease of the fluorescence intensity during the prerecording period before the hypotonic shock. The myofibers which remained intact after treatment with 3 mM CDX, however, did not show any additional increase in water permeability as compared with the myofibers treated with 2 mM drug concentration.

### 3.3. The Existence and Localization of Influenza HA Microdomains Are Not Dependent on the Association with Rafts

**Figure 6:**
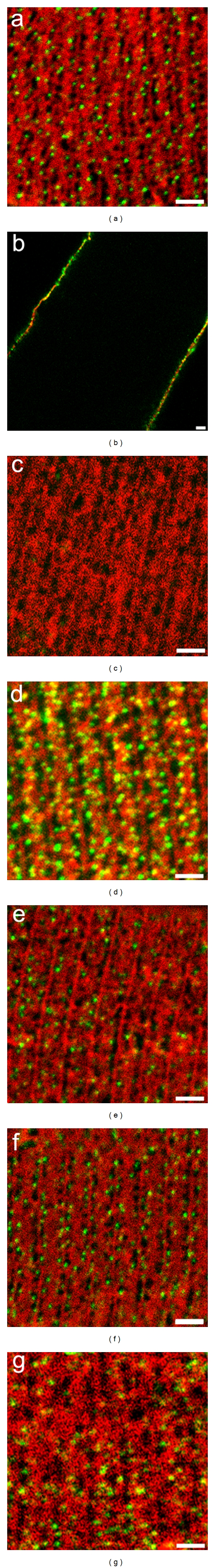
HA localizes to the DGC-deficient domains independently of raft association. Double immunofluorescence staining for the sarcolemmal wild-type HA (green) and *β*-dystroglycan (red) is shown at the sarcolemma level (a) and at the middle of the fiber (b). Double immunofluorescence staining for a noninfected control shows no HA (c). At high expression level, HA occupies regions of *β*-dystroglycan (DGC-rich domain) (d). Treatment with 5 mM CDX does not change the distribution pattern of wild-type HA and *β*-dystroglycan (e). The distribution pattern of the nonraft-associated mutant HA lacking the palmitoylation sites and transmembrane raft-targeting signals also remained unchanged as shown by double staining for *β*-dystroglycan (f). Primary antibody incubation was performed at 10–12°C for 1.5 h before the fixation with 3% paraformaldehyde (a–f). Fixation of the myofiber before application of the anti-HA antibodies did not change the distribution pattern (g). The pictures have undergone brightness and contrast adjustment. Scale bar, 2 *μ*m.

The influenza HA is a viral model glycoprotein that was shown to acquire insolubility in detergents upon travelling from the Golgi apparatus to the plasma membrane [24]. Recent superresolution microscopy observations show that HA at the plasma membrane associates with microdomains ranging from 40 nm to irregularly shaped clusters of up to several microns [12]. Here, we utilized the influenza HA and its variants that are excluded from rafts, in order to further analyze whether raft association played a role in protein targeting to the sarcolemmal domains. We first expressed the raft-associated native HA in isolated myofibers and analyzed its localization on the sarcolemma in relation to the DGC. In order to avoid interference caused by influenza virions that bind to the sarcolemma and the interactions of the HA with other viral proteins, we expressed HA by using recSFV instead of the parent influenza virus. We found that during the propagation of the infection, the HA protein appeared as small dots of uniform size. Some dots located in the domains devoid of DGC, but the majority located to the domain borderlines thus flanking the DGC domains (Figure 6(a)). Accordingly, this localization pattern is clearly distinct from that of caveolae, and it also seems different from that of flotillin 1 that exhibited clusters of irregular shapes and had a tendency to mark the central areas of the domains lacking DGC.

Since the anti-HA antibodies were applied to live myofibers, it is possible that the dots seen were induced by antibody cross-linking. To exclude this possibility, we performed fixing with paraformaldehyde followed by immunofluorescence staining without permeabilization. Fixation prior to the application of antibodies slightly permeabilized the sarcolemma and caused some background staining; however, dots of HA similar to those seen when antibodies were applied before fixation were observed (Figure 6(g)), locating to the DGC-deficient areas but flanking the DGC areas. Upon further propagation of the infection there appeared HA dots also within the domain occupied by the DGC (Figure 6(d)). This was only seen in myofibers exhibiting very intense fluorescence, suggesting overexpression conditions.

In order to approach the question whether it was the association with rafts that determined the localization of HA, we first treated the myofibers with CDX to deplete cholesterol. This treatment had no effect on the distribution of the protein (Figure 6(e)). Next we expressed the variant HA (2A511) that lacked the raft-targeting signals, namely specific hydrophobic amino acids in the transmembrane domain [19]. This mutant has been shown to be detergent soluble in myotubes [18]. As the wild-type HA, the variant HA was expressed using recSFV, and it was found to appear as dots and to localize to the domain borderlines. Finally, we expressed the 2A511 mutant that also lacked palmitoylation sites [7] and found it to behave like the native HA (Figure 6(f)). We did not observe any difference in the dot size between the raft-associated and nonraft-associated forms of HA. Taken together, these findings imply that lipid environment had no crucial role in the targeting of HA.

## 4. Discussion

Previous studies have indicated that several sarcolemmal proteins reside within the DGC domains of the sarcolemmal mosaic [2, 4–6]. Here, we analyzed the distribution pattern of proteins which are associated with sphingolipid- and cholesterol-based microdomains, called rafts, and whether the raft association dictated their distribution patterns. When investigating the localization of three selected raft proteins on the sarcolemma, we found three different types of organization patterns which were dependent on the organization of the DGC. The caveolae rafts harboring cav 3 resided within the DGC domains, and their integrity was strictly dependent on cholesterol. There were clusters of raft proteins, namely flotillin 1 and HA also in the domains lacking DGC. In this respect, the situation resembles that in the polarized epithelial cells in which there are rafts both in the apical as well as the basolateral domains [25–27]. It seems that the integrities of the flotillin and HA protein clusters were not dependent on the association with a lipid-based raft platform.

The omega-shaped invaginations called caveolae comprise a special type of rafts. In skeletal myofibers, these caveolae are located at the sarcolemma and colocalize with the DGC at I-band regions [4]. That the caveolae structures and the localization of cav 3 were dependent on cholesterol is explained by the fact that cav 3 is a cholesterol-binding protein. Abolishing this interaction results in increased mobility of the protein, and it was shifted into the transverse tubules. Previous studies have localized small amounts of cav 3 in the transverse tubules [28]; however, the antibodies we used did not recognize this component. We presume that relatively large amounts of cav 3 shifted to the transverse tubules upon cholesterol depletion whereby it became recognizable to the present antibodies. The fact that cholesterol depletion did not totally redistribute cav 3 is compatible with the idea that this protein is partially bound to DGC via *β*-dystroglycan [29]. A curved membrane may bring cav 3 in the vicinity of the membrane surrounding the caveolae, allowing the two proteins to interact in this restricted area. It is also important to note that the cholesterol depletion did not change the distribution pattern of *β*-dystroglycan although this protein has been previously suggested to reside in rafts [30].

A recent study has posed that the cav 3 protein residing in caveolae also localizes to the DGC-deficient areas at the necks of the transverse tubules [31]. Our results are at variety with this finding since cav 3 appeared to accumulate into transverse tubule necks upon cholesterol depletion only. Instead, we found that flotillin 1 that structurally resembles caveolins was exclusively located in the DGC-deficient membrane domains. Interestingly, there are caveolae-like flasks attached to the necks of the transverse tubules protruding to the sarcolemma [4], and our results suggest that flotillin 1 is present in those structures. Accordingly, electron microscopic immunogold labeling experiments (Figure 1(j)), although suffering from low labeling intensity, support the idea of flotillin 1 locating to the flask structures at the transverse tubule openings. In mononucleated cells, flotillin 1 forms flask structures together with flotillin 2 [21]. Flotillin 2 is not expressed in skeletal muscle cells [32]; however, there may still be an unknown partner for flotillin 1 to form flask structures. It is notable that the glycophosphatidylinositol-anchored carbonic anhydrase IV was recently localized to transverse tubule openings in mouse skeletal myofibers [33], and it is possible that this protein and flotillin 1 reside in the same lipid rafts.

We found that the distribution patterns of flotillin 1 and HA did not change upon cholesterol removal. This is interesting since the organization of rafts and the spatial distribution of some raft-associated proteins are crucially dependent on cholesterol in mononucleated cells. Accordingly, removal of cholesterol has been shown to increase the mobility of raft-associated membrane proteins and to disrupt the organization of microdomains harboring proteins anchored by glycophosphatidyl inositol, resulting in intermixing of different types of rafts [13, 14, 34, 35]. It is notable that cholesterol depletion did not markedly render flotillin 1 soluble in Triton X-100, suggesting an especially rigid lipid organization such as suggested by Ilangumaran and Hoessli [36]. Alternatively, flotillin-1 was bound to subsarcolemmal structures which retain the microdomains in position even upon cholesterol depletion. Our results show that, in addition to the changes in the morphology of caveolae, the barrier function of the plasma membrane is altered as manifested by increased water permeability when cholesterol level is lowered.

Segregation of proteins in liquid-ordered microdomains takes place by means of several known raft-targeting signals [7, 8, 19]. It is possible that removal of these signals leads to diverse distribution patterns of raft-associated proteins on the cell surface. Indeed, it has been demonstrated that raft proteins are transported to the plasma membrane in specific transport vesicles in epithelial cells and also in yeast [37, 38]. We used the prototype raft protein influenza HA and found that HA appeared as small dots, the size of which apparently was below the resolution limit (<200 nm) of the confocal microscope. The dots located to the borderlines of the sarcolemmal domains, but during overexpression conditions, there were low-intensity dots also within the DGC domains. Compatible with this, a previous study showed that influenza virions were budding at the DGC domains during influenza virus infection that typically results in overexpression [4]. It is notable that removal of all known raft-targeting signals from HA did not change its distribution pattern. Importantly, the dot-like appearance of HA did not change, either. It thus seems that HA molecules form small aggregates both in raft and nonraft environments, and these locate to the DGC borderlines, and their size is not dependent on the lipid environment. This idea is compatible with findings of the recent study in which the clustering of the transmembrane domains of HA was found to take place independently of raft association [39]. Regarding the localization, it is possible that anchoring to the subsarcolemmal cytoskeleton plays a role. It is notable that the native transmembrane proteins aquaporin 4 and Na,K-ATPase are associated with the DGC domains via binding to subsarcolemmal *α*-syntrophin and ankyrin-3, respectively [6, 40, 41]. Taken together, we have shown that raft proteins are not evenly distributed in the sarcolemma but locate in discrete regions within the domains defined by DGC. However, the role of the lipid platform in dictating the domain-specific distribution of the proteins varied. It remains to be determined whether the binding of the cytoplasmic tails to the subsarcolemmal cytoskeleton was responsible for the domain-specific partitioning.
